# Establishment of CRISPR-Cas9 system in *Bifidobacteria animalis* AR668

**DOI:** 10.1186/s12934-023-02094-2

**Published:** 2023-06-12

**Authors:** Jiao Li, Xin Song, Zhiqiang Xiong, Guangqiang Wang, Yongjun Xia, Yijin Yang, Lianzhong Ai

**Affiliations:** Shanghai Engineering Research Center of Food Microbiology, School of Health Science and Engineering, University of Shanghai for Science and Technology, 200093 Shanghai, China

**Keywords:** *Bifidobacteria*, CRISPR, Gene knockout, Inducible plasmid curing

## Abstract

*Bifidobacteria* are representative intestinal probiotics that have extremely high application value in the food and medical fields. However, the lack of molecular biology tools limits the research on functional genes and mechanisms of *bifidobacteria*. The application of an accurate and efficient CRISPR system to genome engineering can fill the gap in efficient genetic tools for *bifidobacteria*. In this study, CRISPR system of *B. animalis* AR668 was established, which successfully knocked out *gene 0348* and *gene 0208*. The influence of different homology arms and fragments on the knockout effect of the system was explored. In addition, the inducible plasmid curing system of *bifidobacteria* was innovatively established. This study contributes to the genetic modification and functional mechanism analysis of *bifidobacteria*.

## Introduction

In recent years, intestinal commensal microorganisms have been widely concerned because of their close relationship with human health and diseases. As a type of symbiotic bacteria in the intestines of humans and mammals, *bifidobacteria* has been shown to have a variety of probiotic effects, including regulating the balance of intestinal flora, improving lactose intolerance, improving immune activity, and inhibiting pathogens [1–3]. These positive effects have prompted it to be used commercially as an active ingredient in probiotic foods for health promotion [4]. Although the functional activity of *bifidobacteria* as probiotics has been widely studied, little is known about its exact molecular mechanism of action, which is the key to scientifically explaining its health benefits.

Since the whole genome sequence of *b. longum* was reported in 2002, more and more *bifidobacterium* genome information has been reported, which greatly improved the understanding of physiology, genetics, and evolution of them with medical and commercial significance, and therefore the study of *bifidobacteria* moved from initial morphology to molecular level. The research on the whole genome could be conducive to fully revealing the physiological and metabolic characteristics of *bifidobacteria*, accelerating the further excavation of important functional genes, and laying the foundation for the screening and application of *bifidobacteria.* As of 2022, there were 98 *bifidobacteria* strains that had completed whole-genome sequencing and submitted to the National Center for Biotechnology Information (NCBI, National Center for Biotechnology Information). However, to utilize these abundant genomic data and prove the precise function of genes, it is essential to exploit effective genetic tools and technologies for *bifidobacteria*. Due to the high nutritional requirements, high sensitivity to oxygen, and complex restrictive modification systems, the development of genetic tools for *bifidobacteria* was hindered [5, 6]. At present, relatively few *bifidobacteria* molecular tools have been developed, which explains why little is known about the genetics of these microorganisms compared to other industrially important bacteria.

In fact, gene knockout is a direct procedure to clearly understand the function of specific coding sequences. Only a few attempts have been made in genome editing of *bifidobacteria*. Some studies have proved that the standard knockout method based on traditional homologous recombination is feasible in *bifidobacteria*, but this requires a sufficiently high transformation efficiency[7, 8]. For example, strict anaerobic conditions are used in the preparation and electro-transformation of competent cells to overcome the oxygen limitation of *bifidobacteria*. Nevertheless, due to the lack of RecBCD protein in most *bifidobacteria* genomes, which are responsible for the main homologous recombination pathway of prokaryotes, the homologous recombination method is time-consuming and inefficient which is not suitable for multiple genetic modifications of the *bifidobacteria* genome. As a widely used conditional knockout system, the Cre-loxP system is of great significance for exploring the phenotype and function of specific genes in the genome. However, when Cre recombinase is integrated into the genome, its insertion position hassome limitations, which may affect the expression of endogenous genes. In conditional knockout, the Cre promoter must be effective and specific, which requires pre-screening of Cre promoter genes with high expression levels [9]. Besides, genetic modification will leave scars on the genome. The CRISPR-Cas system has been widely used in the genetic modification of various organisms in recent years because of its precise, fast, and traceless operation [10]. The classic CRISPR-Cas9 technology generates a DNA double-strand break (DSB) at the target site, inducing intracellular homologous recombination (Homology-directed repair, HDR) and non-homologous end joining (NHEJ) repair pathway to achieve genetic modification such as site-specific knockout, replacement, and insertion of genomic DNA[11]. In lactic acid bacteria (LAB), CRISPR-Cas9 gene editing technology can be used to clarify and enhance beneficial characteristics. Oh et al. combined CRISPR-Cas9 with recombination technology in the genetic engineering of *Lactobacillus reuteri* [12]. This is the first time that CRISPR technology has been applied to the genetic engineering of LAB. Research by Leenay et al. showed that CRISPR system could be used for genetic modification of *L. plantarum* WCFS1 and successfully site-directed mutation from D480Y to *rpoB* [13]. Subsequently, Huang et al. established a RecE/T-assisted CRISPR genome editing toolbox that could be used in *L. plantarum* WCFS1 and *L. brevis* ATCC367, which easily achieved gene knockout in *Lactobacillus* [14]. Song et al. established a gene editing plasmid pLCNICK for *Lactobacillus casei* based on CRISPR-Cas9^D10A^. The independent gene knockout and green fluorescent protein (eGFP) gene insertion efficiency of this system was 25–62%, which proved that the pLCNICK gene editing system could be used as a genome editing tool for *L. casei* [15]. In recent years, CRISPR technology has also been applied to anaerobic microorganisms. Cobb et al. applied the CRISPR-Cas9 system to *Streptomyces* for the first time [16]. Huang et al. established a CRISPR system for rapid genome editing of *Clostridium ljungdahlii*, with a gene knockout efficiency of 50–100%, which overcame the lack of genetic tools available for anaerobic *Clostridium* [17]. The successful application of CRISPR technology in LAB and anaerobic microorganisms prompted us to apply it to *bifidobacteria.*

A strain of *B. animalis* called AR668 was screened from baby feces in our laboratory, and its most suitable electro-transformation method through preliminary experiments was established. Based on this, it is planned to establish CRISPR system for *B. animalis* AR668, which will provide a tool platform for better elucidation of its probiotic mechanism and help to systematically elucidate the physiological and metabolic mechanisms at the molecular level that speed up the transformation and breeding of excellent strains.

## Materials and methods

### Strains

*Escherichia coli* Top10 was used as a cloning host. All *E. coli* carrying pAM1 series plasmids were cultured on LB plates containing ampicillin at 37 °C. *Bifidobacterium* AR668 was cultured anaerobically in BS medium at 37 °C for 48 h. If necessary, erythromycin was added at a concentration of 5 µg/ml. All recombinant plasmids enter the competent cells of *bifidobacterium* by electroporation.

### Enzyme and biotechnology toolkit

Phanta Max Super-Fidelity DNA Polymerase and 2× Taq Master Mix (Novozymes Kunming, China) are used for high-fidelity DNA amplification and PCR screening of the required genotypes. PCR procedure was carried out according to the instructions: 95 °C for 3 min, followed by 30 cycles consisting of denaturation at 95 ℃ for 15 s, annealing at 55 ℃ for 15 s, and extension at 72 ℃ for 2 min, and final extension at 72 °C for 5 min. The extension time is appropriately adjusted according to the length of different target segments. The oligonucleotide primers used were synthesized by BGI (Shenzhen, China). Use conventional restriction enzymes and ligases purchased from Takara, Dalian, China to construct plasmids, or use ClonExpress MultiS one-step cloning kit (Vazyme Biotech, Nanjing, China) to assemble plasmids. Plasmid isolation and DNA purification using kits purchased from Axygen (Hangzhou, China).

### Plasmid construction

The plasmids and primers used are shown in Tables 1 and 2. The CRISPR-Cas9 editing plasmid consists of the following parts: the linearized vector is obtained by double digestion of pAM1-ldh2 with *Pst*I and *Spe*I; the Cas9 gene is obtained by PCR amplification with Cas9-*Spe*I-F and Cas9-*Spe*I-R using plcp_*0537* as the template; the whole genome of AR668 is used as the template, PCR amplification of 0348-sgRNA-F and 0348-sgRNA-R was used to obtain 20 bp of the target *gene 0348*; the AR668 whole genome was used as a template, and primers of 0348-up and 0348-down were used for PCR amplification to obtain the homology arms on both sides of the target gene. Note that all 20 bp need to be aligned with the AR668 genome in advance to avoid off-target due to the presence of highly similar sequences.

**Table 1:** Strains and plasmids used in this study

Strains and Plasmids	Characteristic	References and Sources
Strains		
*E. coli* TOP10	Cloing host	Our laboratory
*B. animalis* AR668		Our laboratory
**Plasmids**		
pAM1-ldh2	pMB1 ori, Amp, Em, repB, Pldh2	Our laboratory
plcp_0537	Cas9, repE, repD, Em, Kan, repA101, sgRNA	Our laboratory
pLJ1	pMB1 ori, Amp, Em, repB, Pldh2, Cas9	This study
pLJ2	pMB1 ori, Amp, Em, repB, Pldh2, Cas9, P23, sgRNA, *ldh* homologous arms(1 kb)	This study
pLJ3	pMB1 ori, Amp, Em, repB, Pldh2, Cas9, P23, sgRNA, *upp* homologous arms	This study
pLJ4	pMB1 ori, Amp, Em, repB, Pldh2, Cas9, P23, sgRNA, *ldh* homologous arms(500 bp)	This study
pLJ5	pMB1 ori, Amp, Em, repB, Pldh2, Cas9, P23, sgRNA, *ldh* homologous arms(250 bp)	This study
pLJ6	pMB1 ori, Amp, Em, repB, Pldh-Cas9, P23-sgRNA, *ldh* homologous arms(150 bp)	This study
pLJ7	pMB1 ori, Amp, Em, repB, Pldh2, Cas9, P23, sgRNA, 3 kb homologous arms	This study
pLJ8	pMB1 ori, Amp, Em, repB, Pldh2, Cas9, P23, sgRNA, 5 kb homologous arms	This study
pLJ9	pMB1 ori, Amp, Em, repB, P*lacZ*, eGFP	This study
pLJ10	pMB1 ori, Amp, Em, repB, Pldh2, Cas9, P23, sgRNA, *ldh* homologous arms(1 kb), P*lacZ*, sgRNA(Em)	This study

**Table 2:** Oligonucleotides used in this study

Oligonucleotides	Sequence(5’-3’)	Description
Cas9-*Spe*I-F	AATACTCGTCAGACATGGGCACTAGTTCAGTCACCTCCTAGCTGACTC	For pLJ1 construction
Cas9-*Pst*I-R	GCCAAGCTTGCATGCCTGCAGATGGATAAGAAATACTCAATAGGCTTAGATATCG	
0348-up-F	CGTCAGACATGGGCACTAGTCTCGAGGACCGCCGTGGATTGCCT	For pLJ2 construction
0348-up-R	AGAAGAGGAATCCGACTTCGTCCGCGTC	
0348-down-F	CGAAGTCGGATTCCTCTTCTCCTCTCTCATGCGTC	
0348-down-R	GGTGCTTTTTTTCGGTCTCGAAGCGCACCG	
0348-sgRNA-F	GCTTCGAGACCGAAAAAAAGCACCGACTCGGTGCC	
0348-sgRNA-R	AATGACAATGATGTTGGGCCCCTGTCGTCCAAGGACCGCGAGTTTTAGAGCTAGAAATAGCAAGTTAAAATAAGGC	
0348-P23-F	CGCGGTCCTTGGACGACAGGGGCCCAACATCATTGTCATTCATATTTTTCATTATATTTGGCCT	
0348-P23-R	TAGGAGGTGACTGAACTAGTCGAAAAGCCCTGACAACCCTTG	
0208-up-F	ACATGGGCACTAGTCTCGAGCGCACCGCGGTCT	For pLJ3 construction
0208-up-R	ACTCTAGTAACTACTAGCATATCGACAAACTGTACCC	
0208-down-F	GATATGCTAGTAGTTACTAGAGTAAGCCTTGTTTGTGT	
0208-down-R	GGTGCTTTTTTTACACGACCGCCTGGG	
0208-sgRNA-F	AGGCGGTCGTGTAAAAAAAGCACCGACTCGGT	
0208-sgRNA-R	ATGACAATGATGTTGGGCCCCCTGATGGACAAAGGTGCCAGTTTTAGAGCTAGAAATAGCAAGTTAAAATAAGGC	
0348-HR500-up-F	CGTCAGACATGGGCACTAGTCTCGAGGCCACTTCACGTCCTACGG	For pLJ4 construction
0348-HR500-up-R	AGAGGAGAAGAGGAATCCGACTTCGTCCGCG	
0348-HR500-down-F	GCGGACGAAGTCGGATTCCTCTTCTCCTCTCTCATGCG	
0348-HR500-down-R	GTCGGTGCTTTTTTTTCTAGAGACGACGATCGCCGACC	
0348-HR250-up-F	GGCACTAGTCTCGAGGGCGCTCGCGCTCG	For pLJ5 construction
0348-HR250-up-R	AGAGGAGAAGAGGAATCCGACTTCGTCCGCGT	
0348-HR250-down-F	GCGGACGAAGTCGGATTCCTCTTCTCCTCTCTCATGCGT	
0348-HR250-down-R	GTCGGTGCTTTTTTTTCTAGACGCGGGCGAAACACG	
0348-HR150-up-F	ACATGGGCACTAGTCTCGAGTTCATCATGTTCCTCGTCTTCGAG	For pLJ6 construction
0348-HR150-up-R	AGAAGAGGAATCCGACTTCGTCCGCG	
0348-HR150-down-F	CGAAGTCGGATTCCTCTTCTCCTCTCTCATGCG	
0348-HR150-down-R	TGCTTTTTTTTCTAGAGTTCGCCCACGACGTACG	
3 K-up-F	ACATGGGCACTAGTCTCGAGTGAAGGCGGCGTTGGC	For pLJ7 construction
3 K-up-R	GGGCGAGCCGTCACTCCCGCATGTCGAGCAC	
3 K-down-F	TCGACATGCGGGAGTGACGGCTCGCCCGG	
3 K-down-R	GGTGCTTTTTTTTCTAGATCGGCGGAGAGCGT	
5 K-up-F	ACATGGGCACTAGTCTCGAGGACCGCCGTGGATTGCC	For pLJ8 construction
5 K-up-R	AGAGGAGAAGAGGAATCCGACTTCGTCCGCGTC	
5 K-down-F	GCGGACGAAGTCGGATTCCTCTTCTCCTCTCTCATGCGTCT	
5 K-down-R	GTCGGTGCTTTTTTTTCTAGACGGTCTCGAAGCGCACC	
pAM1-gfp-F	CTGTTTGGAGATCCTTTACTTGTACAGCTCGTCCATGC	For pLJ9 construction
pAM1-gfp-R	GGCAAAGGAGACGGCAAGCTTGATGGTGAGCAAGGGCGAG	
pAM1- *lacZ*-F	TCCTCGCCCTTGCTCACCATCAAGCTTGCCGTCTCCTTTGCCTCC	
pAM1- *lacZ*-R	GACTGGAAAGCGGGCAGTGAATAACTTCACTAGTATAGCGTGCGGG	
sgRNA-Em-F	CGTCAGACATGGGCACTAGTCTCGAGAAAAAAAGCACCGACTCGGTGC	For pLJ10 construction
sgRNA-Em-R	GGCAAAGGAGACGGCCTGTAGTTTTGCATAATTTAGTTTTAGAGCTAGAAATAGCAAGTTAAAATAAGG	
sgRNA-Em-*lacZ*-F	TATGCAAAACTACAGGCCGTCTCCTTTGCCTCC	
sgRNA-Em- *lacZ*-R	AATCCACGGCGGTCCTCGAGGCGGCCGCATAACTTCACTAGTATAGCGTGCGGG	
0348-yz-F	GGTGGACGACACGCCATG	*gene 0348* knockout verification
0348-yz-R	GGAGCTCGGGCGGCC	
0208-yz-F	TAGGCTGCGCGCGTG	*gene 0208* knockout verification
0208-yz-R	CCGCGCATCATCGACATACAG	
3000yz-F	GGTTCGCGCCACACGG	Knockout 3000 bp verification
3000yz-R	GGGGCACCACGTCGATC	
5000yz-F	CTCTGTGGAACATCGTCACCACA	Knockout 5000 bp verification
5000yz-R	GTCGATGCGCTGGCCATC	

The sgRNA and artificially synthesized promoter P23 are assembled by overlap PCR to obtain the sgRNA expression cassette. The above fragments are assembled by two rounds of overlap PCR, and the vector and fragments are assembled by a seamless cloning kit. When preparing a knockout plasmid targeting *gene 0208*, use pLJ2 as a template, and replace the corresponding homology arms on both sides and 20 bp.

Similarly, knockout plasmids with different homology arms are also constructed based on pLJ2, and the homology arms of the corresponding size are replaced with the other originals unchanged. When constructing knockout plasmids with different knockout fragments, only the sequences of the homology arms on both sides of pLJ2 need to be changed.

Construction of the inducible reporter plasmid pLJ9: The linearized vector was obtained by double digestion of pAM1-ldh2 with *Nde*1 and *Spe*1. The promoter sequence of β-galactosidase in the AR668 genome was amplified by pAM1- *lacZ*-F and pAM1- *lacZ*-R, and amplified by pAM1-gfp-F and pAM1-gfp-R. The eGFP sequence of pAM1-ldh2-eGFP was increased, the above fragments were connected by overlap PCR, and the vector and fragments were connected by the seamless cloning kit to obtain the recombinant plasmid pLJ9.

The inducible plasmid elimination system consists of the corresponding knockout plasmid and the inducible sgRNA-Em expression cassette. The linearized vector is obtained by single-enzyme digestion of pLJ2 with *Xho*1 at 37℃ for 3 h, and the *lacZ* sequence of pAM1-*lacZ*-eGFP is amplified by sgRNA-Em-*lacZ*-F and sgRNA-Em-*lacZ*-R, designed to target Em. SgRNA-Em is amplified by sgRNA-Em-F and sgRNA-Em-R, and the two ends are connected to form an inducible sgRNA expression cassette by overlap PCR. The vector and fragments are assembled by seamless cloning kit to construct recombinant plasmid pLJ10.

### Competent cell preparation and electroporation

The constructed plasmids were introduced into AR668 by electroporation. The competent cells were prepared as follows. One milliliter of overnight cultures in BS broth were diluted into 50 ml of fresh BS broth supplemented with 0.5 mol/l sucrose. The inoculated 50 ml BS broth containing additives was anaerobically incubated at 37℃ until the OD_600nm_ reached 0.3. When the cells were cooled on ice for 30 min and harvested by centrifugation (5000 rpm/min, 10 min, 4℃), the supernatant was discarded, the bacterial precipitate was resuspended with the washing buffer (0.5 mol/l sucrose, and 1 mmol/l ammonium citrate), then the supernatant was removed by centrifugation, and the operation was repeated twice. Finally, the cell pellet was resuspended in 1 ml of ice-cold buffer with 10% glycerol. One hundred-microliter aliquots of the competent cells were stored at -80 °C.

The competent cells were mixed with 1000 ng of plasmid DNA and stored on ice for 30 min. Then the mixture was transferred to a pre-cooled Gene Pulser cuvette (Bio-Rad, Hercules, CA, USA). The cuvette was pulsed at various field strengths and parallel resistances using the Gene Pulser Xcell Microbial Electroporation System (Bio-Rad). Following the electroporation, 0.9 ml of BS broth (supplemented with 0.4 mol/L sorbitol, 2 mmol/L calcium chloride and 20 mmol/L magnesium chloride) was added to bacteria and incubated at 37℃ for 4 h in an anaerobic incubator. Then spread the bacteria on BS agar with 5 µg/ml Em. The plates were incubated for 48–60 h at 37 ℃ in an anaerobic incubator.

### Screening and identification of edited genes

The mutants were screened by PCR, and the primers on both sides of the upper and lower homologous arms were designed as verification primers. The PCR-correct strains were further identified by sequencing.

### Evaluation of homology arms and knockout fragments in the CRISPR system

In this study, plasmid pLJ2 was used as an example. On this basis, knock-out plasmids with upper and lower homology arms of 1000 bp, 500 bp, 250 bp, and 150 bp were constructed, which were electrically transformed into AR668, and incubated at 37 °C for 48–60 h. The knockout result of the plasmid was verified, and the result was further confirmed by sequencing.

In this study, the plasmid pLJ2 was taken as an example. On this basis, knockout plasmids with knockout fragments of 1000 bp, 3000 bp, and 5000 bp were constructed, electrotransformed into AR668, and incubated at 37 °C for 48–60 h. Besides the verification of the results, the results were further confirmed by sequencing.

### Establishment of eGFP-based inducible reporting system

A fluorescent reporter plasmid containing eGFP was constructed. Based on plasmid pAM1-ldh2, a vector was prepared with *Hind* III and *Nde* I as double restriction sites, *lacZ* in the AR668 genome was used as a promoter, and eGFP was used as a fluorescent reporter gene to construct a plasmid. The plasmid was transformed into AR668 by electroporation, and a single colony was picked for PCR verification. The single colony containing the correct plasmid was grown in fresh BS broth for lactose induction. The fluorescence is detected by a fluorescence microscope and the relative fluorescence intensity is measured with a microplate reader. The best induction conditions were obtained by optimizing the OD_600nm_ value before induction, the concentration of lactose and the induction time.

### Mutant purification and plasmid elimination for continuous genome engineering

The mutant strains confirmed by PCR and sequencing were cultured in a resistant culture medium. For double-banded strains, that is, when the wild type and the mutant type are present at the same time, they should be streaked and separated in the solid medium first, and then the single-banded strains will be verified by colony PCR. The knock-out strains were cultured in non-antibiotic medium. When it reaches the appropriate OD_600nm_, a certain amount of lactose is added for induction. Under the guidance of the sgRNA targeting the erythromycin resistance gene in the plasmid, Cas9 cuts the erythromycin gene to complete the elimination of the plasmid.

## Results

### CRISPR-Cas9-mediated bifidobacteria genome editing

**Fig. 1:**
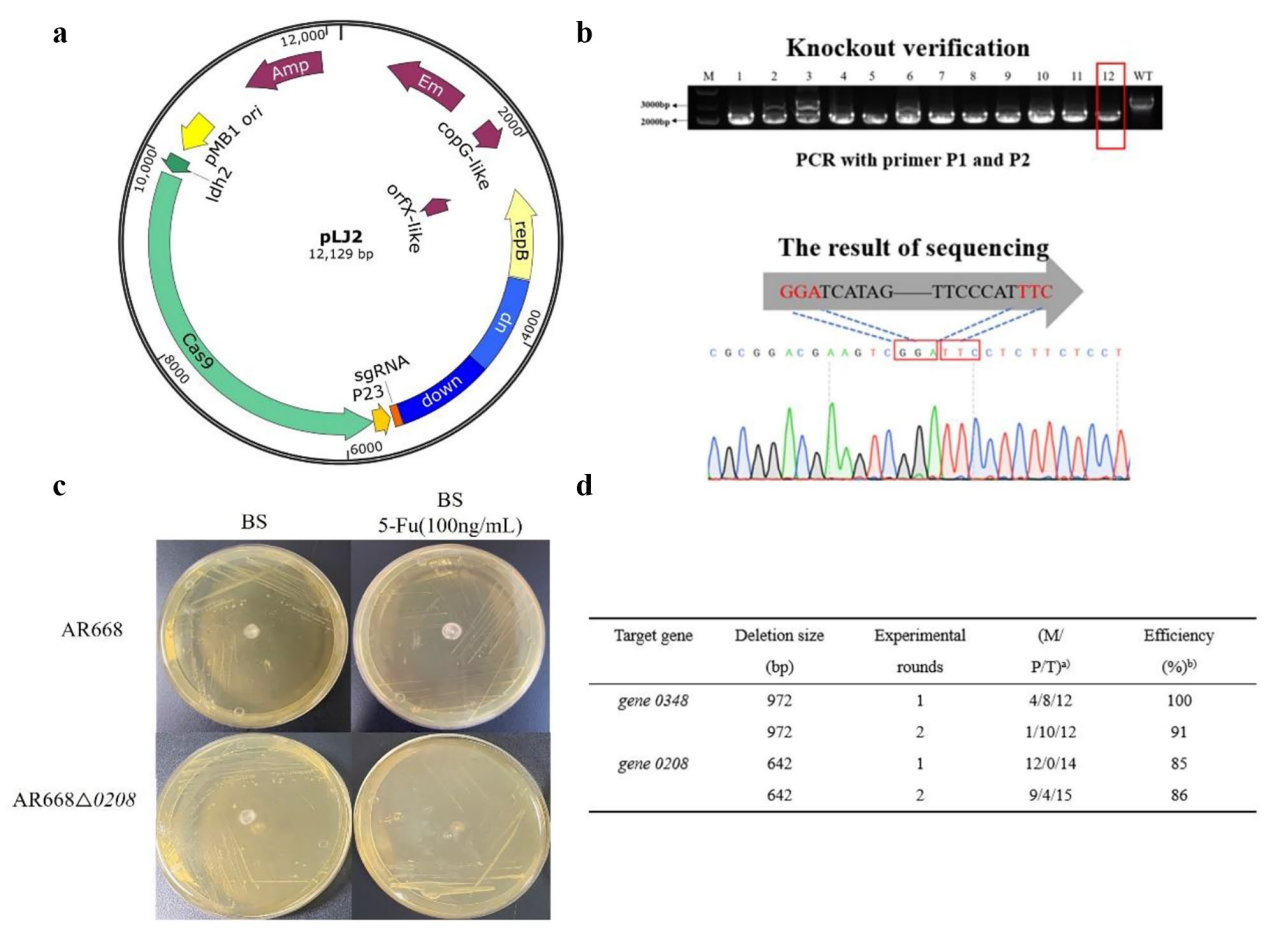
CRISPR gene knockout process and results. (**a**) The map of plasmid pLJ2. (**b**) PCR verification and sequencing results of gene 0348 knockout. (**c**) AR668△0208 mutant and wild-type susceptibility test to 5-fluorouracil. (**d**) Summary of gene knockout in AR668. ^a^) M: number of colonies that harbored mixed wild-type and mutant cells; P: number of colonies that harbored pure mutant cells; T: total number of colonies used for PCR screening; ^b^) Efficiency: probability of deletion events occurring, calculated as (M + P)/T×100%.

In the process of CRISPR-Cas9 gene editing system, DSB is caused by the double-strand break of Cas9 protein at the target site guided by sgRNA. Next, the HDR pathway is needed to repair the broken genome and finally gene knockout is achieved. Cas9 protein has a certain toxic effect on cells, so we first constructed a shuttle plasmid pLJ1 containing the Cas9 expression box and transformed it into *bifidobacteria* by electroporation. We observed the transformation efficiency of pLJ1 containing cas9 protein was (1.33 ± 0.33) ×10^2^ CFU/µg DNA (data is not shown). This proved that Cas9 protein could be expressed in Bifidobacterium with low toxicity. To prove the guided cleavage activity of sgRNA in pLJ2, we constructed a recombinant plasmid pLJ2-1 containing only Cas9 expression box and sgRNA expression box, lacking homology arms. After transformation, only a few colonies appeared (the results of three parallel experiments showed that the number of single colonies produced by pLJ2-1 was 1,1,2, respectively). This means that the designed sgRNA could be used as an element of the CRISPR system. It leads the cleavage of Cas9 protein to cause DSB, and the loss of homologous arms destroys the genome and caused cell death. We constructed a recombinant plasmid pLJ2 targeted *gene 0348*, containing homology arms and sgRNA expression box. After it transformed into *bifidobacteria*, colony PCR verification and sequencing proved that almost all the obtained colonies were mutants. The result had shown that the system successfully knocked out *gene 0348* (Fig. 1).

In addition, the recombinant plasmid pLJ3 targeting *gene 0208* was also constructed. Similarly, when the recombinant plasmid was introduced into *bifidobacteria*, the knockout efficiency from obtained transformants was 80%. The gene 0208 (gene *upp*) encodes the uracil phosphoribosyltransferase. This gene widely exists in prokaryotes and some lower eukaryotes, and its expression product is uracil phosphoribosyltransferase (UPRT). UPRT can convert 5-fluorouracil into 5-fluoromonophosphate deoxyuracil, which can inhibit the activity of thymidine synthase and lead to cell death. Thus, the upp-deleted strain can grow on the medium containing 5-fluorouracil (Fig. 1). Generally speaking, we have constructed a CRISPR plasmid that can be used for *bifidobacteria* gene knockout.

### Exploring the smallest homology arm of the CRISPR system

Next, we explored the effect of different homology arms in recombinant plasmids on the knockout efficiency. On the basis of pLJ2, recombinant plasmids pLJ4, pLJ5, and pLJ6 with homology arms of only 500 bp, 250 bp, and 150 bp were designed, and the other elements were unchanged. After these plasmids electroporation into *bifidobacteria* respectively, the knockout effect was observed in the Table 3. When the homology arm was 1000 bp, the system had the highest ability for *gene 0348*, and when the homology arm was 500 bp, the knockout efficiency was>80%. In a word, the system had a quite good knock-out ability, even if the homology arm was 150 bp with a knockout efficiency of more than 50% (Table 3). But the efficiency of pure gene-deleted was low which may be due to imperfect homologous recombination ability or insufficient culture time.

**Table 3:** The effect of homology arms size and target fragment size on knockout

Influencing factors	Size (bp)	Number of experiments	Results		
			(M/P/T)^a^	Efficiency (%)^b^	Efficiency of pure gene-deleted (%)
Homology arms	500	1	9/0/10	90	0
		2	8/3/13	84	23
	250	1	7/0/12	58	0
		2	9/1/16	62	6
	150	1	6/2/14	57	29
		2	5/2/16	44	12
Target fragments	3000	1	3/0/15	20	0
		2	3/0/13	23	0
	5000	1	0/0/8	0	0
		2	0/0/12	0	0

^a^) M: number of colonies that harbored mixed wild-type and mutant cells; P: number of colonies that harbored pure mutant cells; T: total number of colonies used for PCR screening;

^b^) Efficiency: probability of deletion events occurring, calculated as (M + P)/T×100%.

### Exploration of the largest knockout fragment of the CRISPR system

Similarly, the maximum knock-out segment of the system was explored. Based on pLJ2, we designed recombinant plasmids pLJ7 and pLJ8 with knockout fragments of 3000 and 5000 bp, while other elements remained. The recombinant plasmids were introduced into competent cells respectively, and the result was shown in the Table 3. When the knockout fragment was 3000 bp, its knockout effect was only about 20%, but the target fragment of 5000 bp can’t be knocked out yet (Table 3).

### Screening and optimization of inducible promoter based on enhanced green fluorescent protein (eGFP)

**Fig. 2:**
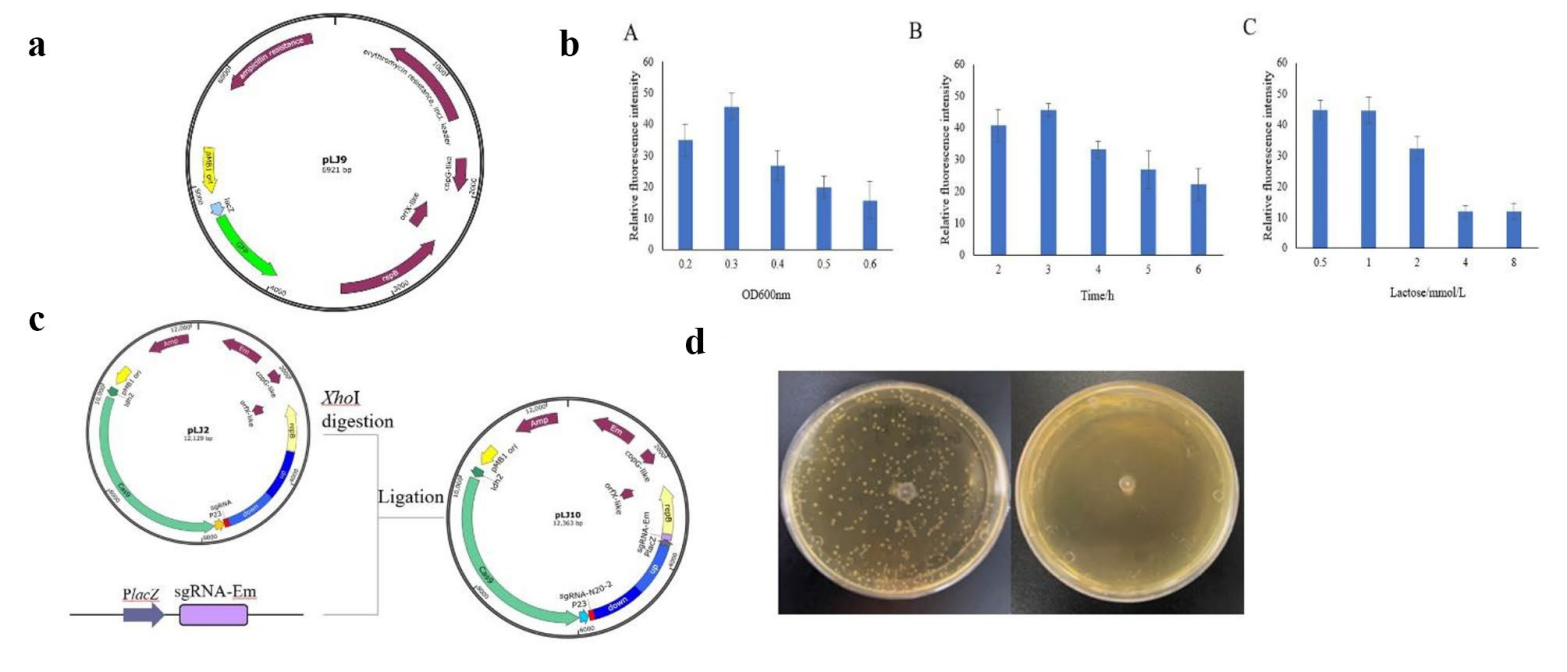
Construction of inducible plasmid curing system. (**a**) The map of plasmid pLJ9. (**b**) The optimization of lactose induction conditions. (**c**) Construction process of inducible plasmid pLJ10. (**d**) After the culture was induced according to the optimized conditions, it was cultured on a resistant plate and compared with the culture that was continuously passaged in a non-resistant medium

The complete genetic operating system included a plasmid curing procedure for subsequent operations. However, the traditional method of plasmid elimination is continuous passage in antibiotic-free culture medium, and the plasmid still existed, which was time-consuming and inefficient. Therefore, this method was not suitable for our system. In this study, the *bifidobacteria* self-inducible promoter was selected to eliminate the recombinant plasmid. The ldh2 promoter in pAM1-ldh2 was replaced with the β-galactosidase promoter P*lacZ* in AR668, and eGFP was inserted to form the recombinant plasmid pLJ9. The plasmid was electroporated into AR668. After the PCR program verification, it was cultured in a liquid medium. After the induction of lactose, the relative fluorescence was measured by a microplate reader. AR668 containing pAM1-ldh2 was the control. By optimizing OD_600nm_ value, induction concentration, and induction time, the optimal induction conditions were 0.3, 1%, and 3 h, respectively (Fig. 2).

### Construction of inducible plasmid curing system

**Fig. 3:**
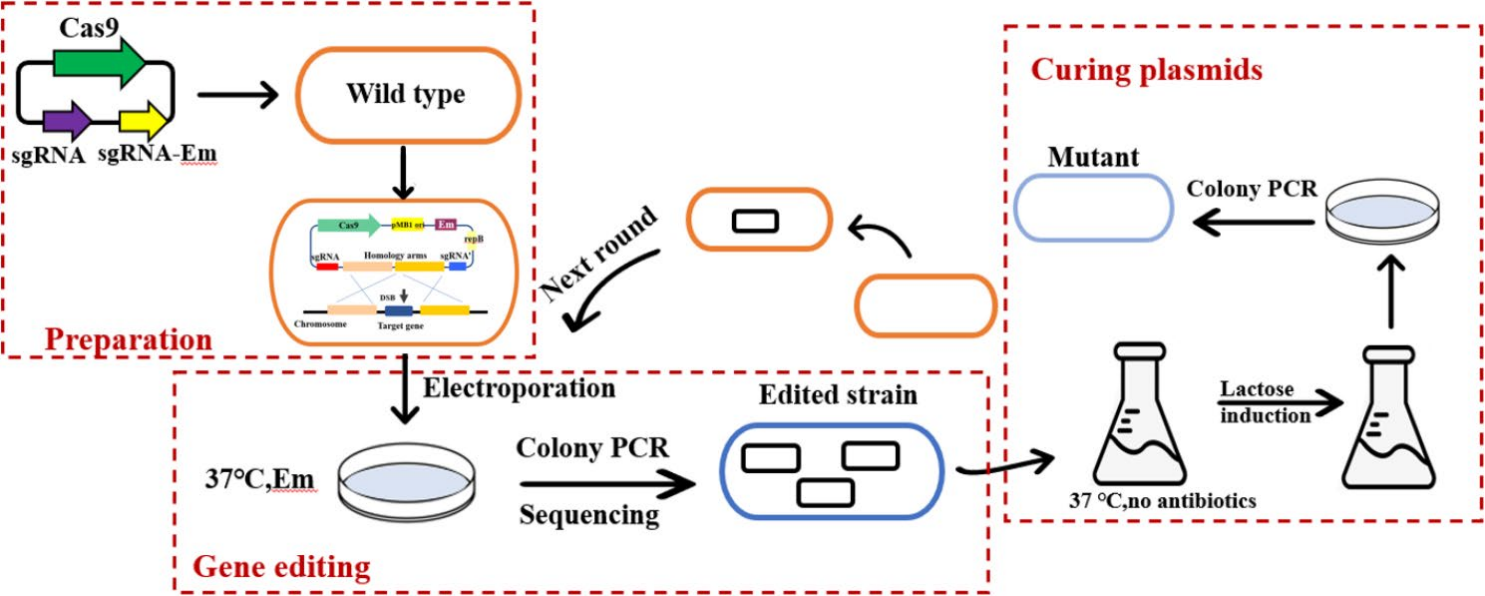
The overview of CRISPR–Cas9 editing toolbox for AR668. The working plasmid contains three parts, the Cas9 expression box, the sgRNA1 constitutive expression box targeting the knockout gene and the sgRNA2 inducible expression box targeting Em gene. The working plasmid is electro-transformed into bifidobacteria, then the target gene is cut by Cas9 protein under the guidance of sgRNA1, and homologous recombination repair can repair the broken genome through to complete gene editing. Next, the plasmid was induced and eliminated by lactose to obtain a pure mutant strain for the next round of genetic modification

Through the optimization of the above conditions, we obtained the most suitable induction conditions. Next, pLJ10 was constructed on the basis of pLJ2, whichcontained sgRNA induced expression cassette. It was worth noting that the second sgRNA targeted erythromycin (Em) in this plasmid. When lactose was not added, P*lacZ* was ineffective. The sgRNA targeting *gene 0348* guided Cas9 to cut the target gene. After induction by lactose, the Cas9 protein cleaves the Em gene with the guidance of the second sgRNA. Without homologous arm repair, the plasmid was broken, thereby completing the plasmid elimination. Figure 2 showed the growth of the strain in the resistant culture medium after the traditional passage and lactose induction, respectively, which successfully verified the feasibility of the plasmid solidification system. The complete gene editing process is shown in Fig. 3.

## Discussion

As a kind of important probiotics, *bifidobacteria* has a wide range of practical value, but its application is limited compared to other industrial microorganisms. The higher nutritional requirements and complex restriction-modification systems undoubtedly leads to difficulties of developing effective molecular manipulation tools [18]. The research on the physiological and probiotic characteristics of *bifidobacteria* is limited. A stable plasmid suitable for *bifidobacteria* and an efficient electrotransformation method are the key prerequisites for genetic modification[19]. Due to the specificity of *bifidobacteria* species, the competent and electrotransformation methods are not universal. After optimization of the preliminary conditions in our laboratory, the electrotransformation efficiency of pAM1-ldh2 in AR668 reached more than 10^5^ CFU/ug DNA, which laid a good foundation for later genetic transformation. Nevertheless, the efficiency of the plasmid transformation of *bifidobacteria* is affected by several factors. In future research, the expression of Cas9 and sgRNA can be controlled by inducible promoters. In addition, the RM system can be artificially modified or targeted mutagenesis of the plasmid. All these operations above can reduce the dependence of the editing efficiency of this system on high transformation efficiency.

With the application of the CRISPR system in LAB and anaerobic microorganisms such as *Clostridium*, the CRISPR system in *bifidobacteria* was explored. There have been reported on the endogenous CRISPR system in *B.longum* [20]. Although the endogenous CRISPR system is more suitable for bacteria, most *bifidobacterium* genome contains a typeI CRISPR system element which its application is more difficult to develop [21]. At present, the typeIICRISPR system is the simplest, but the expression of Cas9 protein and its toxicity to cells need to be considered [22]. This study has proved that the Cas9 protein from *Streptococcus pyogenes* can be expressed and exerted its effect in AR668. In this study, an exogenous CRISPR system suitable for *bifidobacteria* has been established, which provided an effective tool for the exploration of the function and probiotic mechanisms of *bifidobacteria*.

The traditional method to cure plasmids is to repeat passage in the resistance-free medium. In this study, the replicon of pAM1 plasmid is derived from the recessive pMB1 of *Bifidobacterium* L48 [23–25] which is considered to be replicated through theta mode with strong stability. We have proved through preliminary experiments that this series of plasmids containing continue to exist in the non-antibiotic medium, which undoubtedly prolongs the working time of the editing system. The suicide gene SacB is widely used in reverse screening, but it is not suitable for this study. This is because the recovery medium contains sucrose to protect the cell wall during the electrotransformation of *bifidobacteria*. Nisin is a food-grade induction system commonly used in LAB, requiring the presence of the RK system [26, 27]. It was originally discovered in *L.lactis* and then widely used. Some studies have proved that the RK system can be heterologous expressed in other LAB such as *L. reuteri* [28]. However, *bifidobacterium* can’t use the nisin system either. The nisin-inducible promoter is not completely unexpressed when no foreign substances are added, but the expression is low and difficult to detect, which will also affect the expression of knockout plasmid in *bifidobacteria*. In this study, a lactose induction system derived from *bifidobacteria* itself was used. The application of this system is more mature on most bacteria, and the use of lactose as an inducer avoids the toxicity of IPTG to cells and the relatively expensive price. The plasmid was knocked out by Cas9 by designing the sgRNA targeting the resistance gene in the plasmid. In future studies, it would be easier to use temperature-sensitive replicons or plasmids that are easily lost in the passage process.

The development of whole genome sequences and genetic tools provides opportunities to improve the analysis of physiology, genetics, and gastrointestinal metabolism in *bifidobacteria*. Through appropriate genetic modification, the survival rate in vivo of probiotics can be improved, and the ingested probiotic strains can be tracked by molecular method in human or animal disease models. Future research needs to optimize the system to expand the scope of application such as exploring the practicability of this system in other *bifidobacteria*, genetically modifying AR668, and selecting mutant strains with excellent traits for industrial large-scale production.

## Conclusion

Taken together, the present study established the CRISPR system of *B. animalis* AR668 for genetic modification and functional mechanism analysis.

## Data Availability

All data have been stored on dedicated computers at the University of Shanghai for science and technology. All data and strains are available upon request.
